# The role of precisely matching fascicles in the quick recovery of nerve function in long peripheral nerve defects

**DOI:** 10.1097/WNR.0000000000000873

**Published:** 2017-09-20

**Authors:** Liwei Yan, Zhi Yao, Tao Lin, Qingtang Zhu, Jian Qi, Liqiang Gu, Jintao Fang, Xiang Zhou, Xiaolin Liu

**Affiliations:** a Department of Microsurgery and Orthopedic Trauma, the First Affiliated Hospital of Sun Yat-sen University; b Center for Peripheral Nerve Tissue Engineering and Technology Research, Guangdong, Guangzhou, People’s Republic of China

**Keywords:** fascicle matching, nerve function recovery, peripheral nerve injury, tissue-engineered nerve grafts

## Abstract

Peripheral nerve injury therapy in the clinic remains less than satisfactory. The gold standard of treatment for long peripheral nerve defects is autologous nerve grafts; however, numerous clinical complications are associated with this treatment. As tissue engineering has developed, tissue-engineered nerve grafts (TENGs) have shown potential applications as alternatives to autologous nerve grafts. To verify the important role of the biomimetic pathway of fascicle design in TENGs, we designed an animal model to study the role of the precise matching of fascicles in the effectiveness of nerve function recovery. 24 Sprague-Dawley rats were divided randomly into three groups (eight/group) that corresponded to 100% fascicle matching (100%FM), 50%FM and 0%FM. We selected Sprague–Dawley rat long-gap (15 mm) sciatic nerve defects. In the 6 weeks after surgery, we found that the 100%FM group showed the most effective functional recovery among the three groups. The 100%FM group showed better functional recovery on the basis of the sciatic functional index than the 50%FM and 0%FM groups. According to histological evaluation, the 100%FM group showed more regenerating nerve fibres. Moreover, in terms of the prevention of muscle atrophy, the 100%FM group showed excellent physiological outcomes. The 100%FM as tissue-engineered scaffolds can enhance nerve regeneration and effective functional recovery after the repair of large nerve defects. The results of this study provide a theoretical basis for future TENG designs including biomimetic fascicle pathways for repairing long nerve defects.

## Introduction

Nerve injuries, especially peripheral nerve injuries (PNIs), are common clinical problems worldwide 1, causing morbidity in over 2.8% of trauma patients 2. Peripheral nerve damage following trauma or neuropathic manifestations often leads to permanent disability through loss of sensory and motor function and an enormous socioeconomic burden 3,4. The most efficient clinical treatment for peripheral nerve defects is graft implantation, such as autografts and acellular nerve allografts (ANAs), which have major advantages of closely mimicking natural nerve characteristics 5. However, autografts and ANAs used to repair long nerve defects have some disadvantages, such as donor nerve distal organ dysfunction, physical and microstructure size mismatching, requirement of surgery at the donor site, limited donor sources and potential immune responses 6,7. This has motivated alternative nerve repair strategies, such as three-dimensional (3D) printed nerve guidance channels, which provide anatomical pathways for the regeneration of damaged nerves 8.

Peripheral nerves are constituted of nerve fibres, which include sensory fibres that receive afferent sensory impulses and motor fibres that send efferent motor instructions, and are among the most important structures 9. The perineurium that encases nerve fibres protects and maintains the pressure of their internal environment 10. In the clinic, we found that in short nerve gaps (<20 mm), end-to-end neurorrhaphy is the best option to restore nerve function by which the perineurium of the proximal stump is connected to the distal stump with the same neural topography 11. For long peripheral nerve gaps (>20 mm), autografts, ANAs and tissue-engineered nerve grafts (TENGs) can be used for repair. Theoretically, the optimal goal of implanting a graft is to improve nerve injury classifications from Sunderland level V to level III 12. Thus, 3D printing nerve grafts for repairing long nerve defects may be beneficial compared with other grafts. Until now, no biofabricated grafts have reached the biomimicry fascicle pathway to achieve end-to-end neurorrhaphy. Our team previously focused on using a biofabricated nerve graft strategy to repair long peripheral nerve defects. However, it is unknown whether precisely matched fascicles in the pathway design are necessary for the use of our 3D printing nerve grafts. Thus, we designed this experiment to verify the important role of precisely matching fascicles in the effectiveness of nerve function recovery in long peripheral nerve defects. The results of this study provide a theoretical basis for next-generation TENGs for repairing long nerve defects.

## Materials and methods

### Animals

In our experiment, we utilized 24 male Sprague-Dawley (SD) rats weighing 250–350 g and provided by Sun Yat-sen University in the Guangzhou of Guangdong Province of China. The Administration Committee of Experimental Animals of The First Affiliated Hospital of Sun Yat-sen University approved and supervised the use of laboratory animals.

### Surgical procedures and experimental groups

**Fig. 1:**
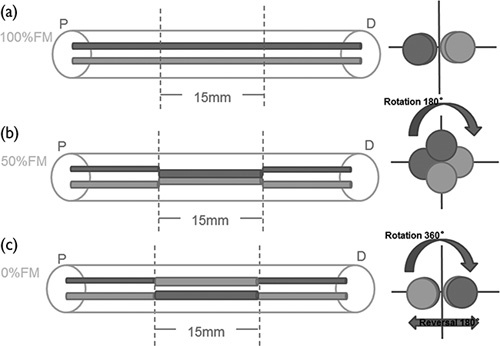
Schematic view of the surgical procedures in each experimental group. (a) 100%FM group; (b) 50%FM group; and (c) 0%FM group. D, distal stump; FM, fascicle matching; P, proximal stump.

Our experiment used a total of 24 SD rats that were randomized into three groups corresponding to 100% fascicle matching (100%FM), 50%FM and 0%FM operating methods. We selected male adult rats and each group included eight rats as described previously 13. The surgical methods were as follows: the same surgeon with microsurgery training for more than 5 years performed all surgical procedures in an aseptic room and ketamine HCl (100 mg/ml) was used for intraperitoneal anaesthesia. The left sciatic nerve was exposed as the experimental side and the right sciatic nerve was exposed as the control side. For all experimental groups, we induced a 15 mm gap. For the 100%FM group, a 15-mm long sciatic nerve was excised and re-planted in the original position. For the 50%FM group, the nerve segment was reimplanted at an axial rotation of 180°. For the 0%FM group, the nerve segment was reimplanted at an axial direction rotation of 360° with a reversal of 180° (Fig. 1). Under the microscope, 8-0 monofilament nylon sutures were used for end-to-end anastomosis.

### Functional evaluation of nerve regeneration

We applied sciatic functional index (SFI) to assess the functional recovery of motor nerves after sciatic nerve injury. The SFI value is a well-established and commonly used method for assessing motor recovery, as previously described 14,15. The SFIs of animals were assessed at 0, 2, 4 and 6 weeks postoperatively. During walking-track analysis, all investigators were blinded to group number to avoid bias.

### Histology

After SFI assessment, we randomized four rats from each group at 2, 4 and 6 weeks for histological examination. The implantation grafts were excised from the rats’ sciatic nerves and cut into three parts. The medial stump was prepared for transmission electron microscope analysis. The specific operation was as follows: the nerve sample was fixed in 4% paraformaldehyde for 1 day and ultra-thin sections (50 nm thick) were cut immediately after a series of semithin sections with the same ultramicrotome (Thermo NX50; Thermo Scientific, Waltham, USA) and collected on a pioloform-coated grid. Sections were double-stained with a saturated aqueous solution of uranyl acetate and lead citrate 16. By electron microscopy, six images were selected using a systematic random sampling protocol of live images at ×5800 magnification. The morphometric parameters measured included axonal diameter, myelin sheath thickness and the *G*-ratio of the myelinated nerve fibres as described previously 17. At the middle of the implant, we harvested a sample (1 mm thick) for toluidine blue staining for analysis of the number of myelinated axons. The distal stump was used to examine structures formed by the new nerve fibres. We used H&E staining to observe longitudinal structures as described previously 13. For histological analysis, nerves were fixed in 4% paraformaldehyde for 2 h, followed by several washes in PBS for 24 h. The fixed nerves were dehydrated by a graded series of ethanol, embedded in paraffin wax, ;longitudinally sectioned, cut using a cryostat (CM3050s; Leica, Wetzlar, Germany) to a thickness of 3 mm and mounted on microscope slides. Sections were stained with H&E to visualize the longitudinal structure of the nerves.

### Immunohistochemical analysis

The distal stump was assessed using the H&E protocol and the last staining steps incorporated antibodies of neurofilament NF-200 and S-100 as markers for axons and myelin sheath. The specific procedures were as follows: the longitudinally sectioned samples were immersed in a 1/200 NF antibody solution (Sigma-Aldrich, St. Louis, Illinois, USA) and 1/200 S-100 protein antibody (Santa Cruz Biotechnology, Dallas, Texas, USA) for 1 h at 37°C. Then, the longitudinally sectioned samples were immersed in biotinylated anti-mouse rabbit IgG solution for 1 h. Horseradish peroxidase-labelled secondary antibody was developed using the diaminobenzidine method. Six images were selected randomly for each sample after NF-200 and S-100 staining. We used ×40 magnification to capture the images of the regenerated nerve area and ×200 magnification to randomly capture images of at least 40% of the regenerated area for analysis.

### Muscle histology

We dissected the triceps surae muscles from tibia bones at 6 weeks. Small pieces of the triceps surae muscle samples (measuring 5×5×5 mm) were immersed in an optimum cutting temperature compound (O.C.T compound; Sakura of America, Hayward, California, USA). Transverse sections were cut using a cryostat (CM3050s; Leica, Wetzlar, Germany) to a thickness of 50 mm and mounted on microscope slides. They were then subjected to Masson trichrome staining and observed under a light microscope. We collected four photographs from randomly selected fields for each specimen and then measured the cross-sectional area of the muscle fibres using Image-Pro plus software (MediaCybernetics, Bethesda, Maryland, USA). The percentage of collagen fibres was calculated by dividing the collagen fibre area by the collagen fibre and the muscle fibre area.

### Statistical analysis

SPSS 24.0 software package for Windows (SPSS Inc., Chicago, Illinois, USA) was used for statistical analysis. The data represent the means values±SD. Student’s *t*-test was used to test the statistical significance of differences between sample means. Values of *P* less than 0.05 and less than 0.01 were considered statistically significant.

## Results

### Intraoperative view of the surgical process

**Fig. 2:**
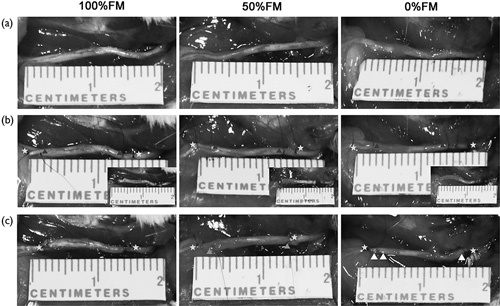
The process of the surgery. (a) Pre-operation. (b) Intra-operation. (c) Post-operation. Three-point star mark the nerve defect points; Four-point star mark the nerve scaffold proximal points; Five-point star mark the nerve scaffold distal points; Six-point star mark the nerve scaffold proximal points rotation 180°; Seven-point star mark the nerve scaffold distal points rotation 180°; Triangle mark the nerve scaffold proximal points rotation 180° and re-implant in distal point; Square mark the nerve scaffold distal points rotation 180° and re-implant in proximal point.

In the operation, we exposed the left sciatic nerves as shown in Fig. 2. None of the left sciatic nerves of the rats showed any swelling, gross inflammatory reactions, deformation or breakage in the peripheral nerve tissue or implants upon careful exposure after 6 weeks.

### Motor function assessment

**Fig. 3:**
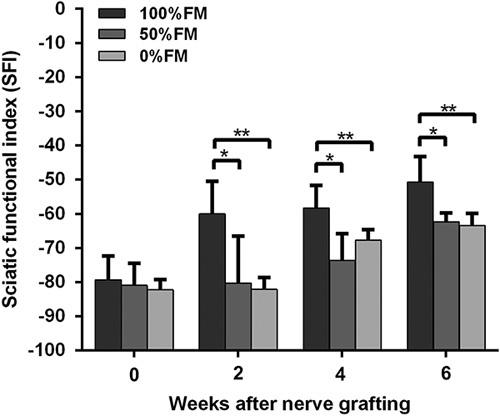
The sciatic functional index (SFI) values of the three groups at 0, 2, 4 and 6 weeks after operation. *n*=8 for each group. *P<0.05, **P<0.01. FM, fascicle matching.

The SFI value, which varies from 0 (normal function) to −100 (complete dysfunction), was calculated to evaluate locomotive functional recovery in rats. The SFI values of the three groups increased with time (Fig. 3). One day after operation, which was defined as 0 week, we evaluated the SFI. After operation, the SFI values of all animals decreased to −80 and no obvious difference was observed among groups. At week 2, the SFI values of the three groups improved markedly. The 100%FM group showed a greater increase in SFI than the other two groups. At 2 weeks, the SFI value of the 100%FM was significantly higher than those of the 50%FM (*T*=2.979, *d.f.*=10 and *P*<0.05) and 0%FM (*T*=5.352, *d.f.*=10 and *P*<0.01) groups. At 4 weeks, the SFI value of the 100%FM was significantly higher than those of the 50%FM (*T*=3.615, *d.f.*=10 and *P*<0.05) and 0%FM (*T*=3.096, *d.f.*=10 and *P*<0.01) groups. At 6 weeks, the SFI value of the 100%FM was significantly higher than those of the 50%FM (*T*=3.636, *d.f.*=10 and *P*<0.05) and 0%FM (*T*=3.803, *d.f.*=10 and *P*<0.01) groups, whereas the SFI value of the 50%FM and 0%FM groups did not differ significantly at 2, 4 and 6 weeks, respectively.

### Histological analysis

**Fig. 4:**
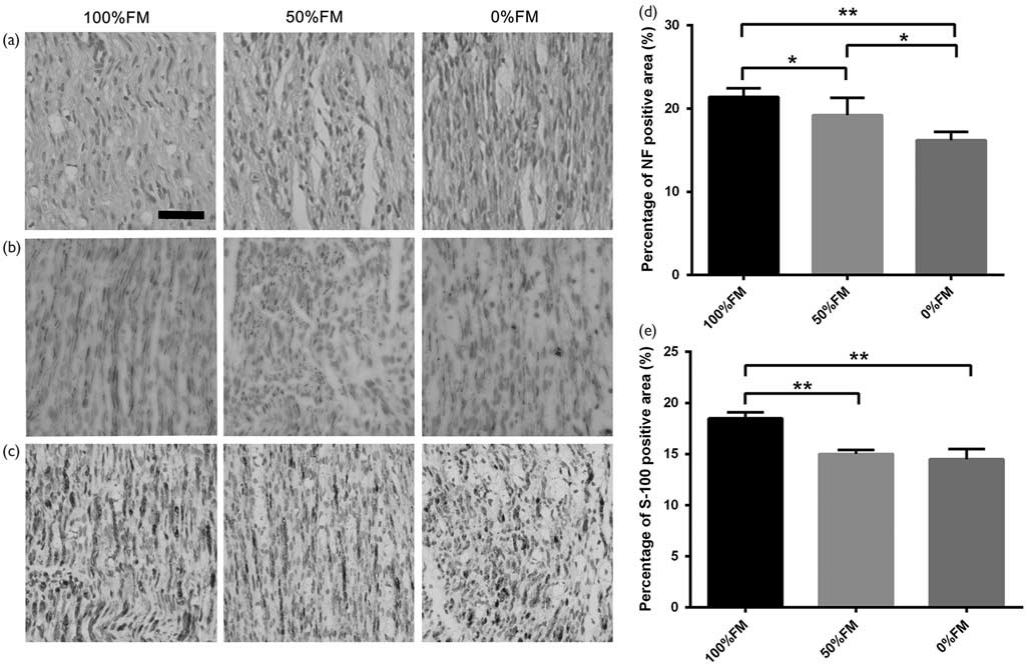
Histological and immunohistochemical analysis of the three groups at 6 weeks after surgery. (a) The longitudinal sections of the distal part after H&E staining. (b) NF-200 antibody immunostaining of longitudinal sections. (c) S-100 Schwann cell marker immunostained longitudinal sections. (d) NF-200 statistical analysis of the three groups. (e) S-100 statistical analysis of the three groups. There were significant increases in the numbers of positively stained neurofilaments and S-100-positive areas for the 100%FM and 0%FM groups. Error bars correspond to the mean±SD (*n*=6 for each groups). *P<0.05, **P<0.01. Scale bar=50 µm. FM, fascicle matching.

Histological evaluation of the distal sections was performed at 2, 4 and 6 weeks after the operation to isolate and evaluate the newly formed nerve fibres of each group. First, H&E staining was used to evaluate the longitudinal sections of the nerves. Linearly ordered structures were observed on the newly formed nerves of the three groups. The H&E staining did not indicate any inflammatory reactions around the regenerated nerves. Among the three groups, we found more newly formed nerves and more correctly ordered linear guidance in the 100%FM group than in the other groups (Fig. 4a). At the injured site, NF-positive-stained axons were analysed to investigate axonal regeneration (Fig. 4b). S-100 staining was used to investigate SC regeneration in the grafts (Fig. 4c). The 100%FM group showed significantly higher NF-positive areas compared with the other groups according to the statistical analysis, especially compared with the 0%FM group (*T*=8.676, *d.f.*=10 and *P*<0.01) (Fig. 4d). The S-100-positive-stained area was also statistically analysed (Fig. 4e) and the result showed that the 100%FM group presented with the highest S-100-positive-stained area compared with the 50%FM group (*T*=8.327, *d.f.*=4 and *P*<0.01) and the 0%FM group (*T*=4.879, *d.f.*=4 and *P*<0.01).

### Remyelination of nerves

**Fig. 5:**
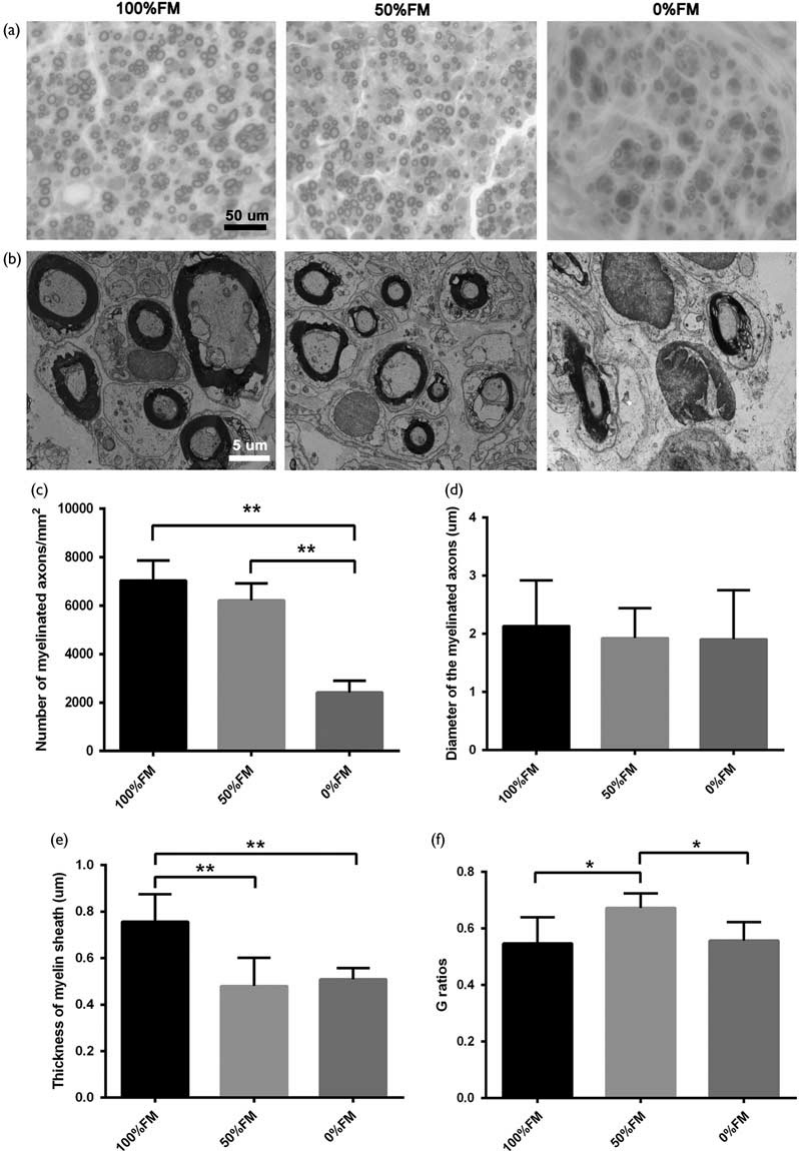
Transverse sections of the middle part of the regenerated nerve at 6 weeks postoperatively. (a) Toluidine blue staining. (b) Transmission electron micrograph (TEM) images of regeneration axons. (c) The number of myelinated axons in each area (mm^2^). The 100%FM and 50%FM groups appeared to have more regenerating nerve fibres than the 0%FM group. (d) The diameter of the myelinated axons from TEM evaluation. (e) Thickness of the myelin sheath. (f) *G*-ratios of the myelinated nerve fibres. Error bars correspond to the mean±SD (*n*=6). *P<0.05, **P<0.01. Scale bar=50 and 5 µm, respectively. FM, fascicle matching.

Toluidine blue staining (Fig. 5a) and transmission electron microscopic (Fig. 5b) of the middle sections were carried out to analyse nerve remyelination among the three groups. Nerve fibres from the 100%FM and 50%FM groups were more regularly arranged than those from the 0%FM group. The number and diameter of remyelinated axons were also evaluated statistically (Fig. 5c and d); however, the diameter of the myelinated axons was not significantly different among the three groups. At 6 weeks after nerve grafting, the 100%FM group had the greatest number and diameter of myelinated axons, followed by the 50%FM and 0%FM groups. For the number of myelinated axons, the 100%FM had significantly more axons than the 0%FM group (*T*=10.886, *d.f.*=8 and *P*<0.01) and the 50%FM also had significantly more axons than the 0%FM group (*T*=9.996 and *d.f.*=8 and *P*<0.01). The thickness of the new myelin sheath was measured (Fig. 5e); the 100%FM had significantly thicker myelin sheath than the 0%FM group (*T*=5.152, *d.f.*=18 and *P*<0.01) and the 50%FM also had significantly thicker myelin sheath than the 0%FM group (*T*=4.47, *d.f.*=13 and *P*<0.01). The *G*-ratio of the 50%FM group was higher than that of the 100%FM group (*T*=3.737, *d.f.*=18 and *P*<0.05) and the *G*-ratio of the 50%FM group was higher than that of the 0%FM group (*T*=3.734, *d.f.*=13 and *P*<0.05) (Fig. 5f).

### Triceps surae muscle evaluation

**Fig. 6:**
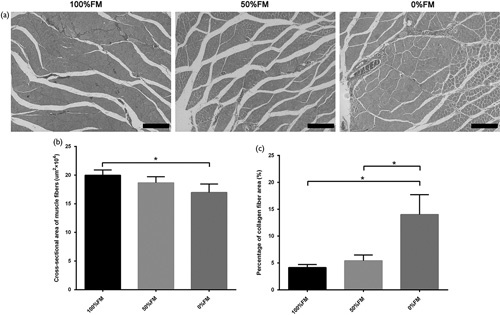
Triceps surae muscle reinnervation. (a) Masson trichrome staining of transverse sections. (b) Cross-sectional area of muscle fibres. (c) Cross-sectional area of collagen fibres. Error bars correspond to the mean±SD (*n*=6). *P<0.05. Scale bar=200 µm. FM, fascicle matching.

We used Masson trichrome staining to determine the target muscle morphometry at 6 weeks postoperatively (Fig. 6a). The outline, reinnervation and cross-sectional area of muscles were evaluated by muscle fibre type. The 100%FM group had larger muscle fibre areas than the 0%FM (*T*=2.969, *d.f.*=4 and *P*<0.05) (Fig. 6b). The average percentage of collagen fibre area in the target muscle on the injured side was significantly larger in the 0%FM group than in the 100%FM (*T*=4.465, *d.f.*=4 and *P*<0.05) and 50%FM groups (*T*=3.793, *d.f.*=4 and *P*<0.05) (Fig. 6c).

## Discussion

Significant progress has been made in the study of PNI and regeneration over the past decades. However, full functional recovery remains hindered because of the lack of reliable biological or synthetic nerve constructs for nerve defect repair 18,19. 3D printing technology has recently successfully shown the capacity for printing customized scaffolds for PNI repair 8,20–22. 3D printing, an additive manufacturing technology, is a powerful tool for fabricating 3D constructs with intricate geometries under computer-aided design/computer-aided manufacture system control 23. The current technique has been applied to manufacture customized medical devices, such as amputee prosthetics, airway splints and scaffolds for tissue engineering 24. The model designs used for 3D printing customized nerve scaffolds are currently derived from medical imaging techniques, such as computed tomography, MRI 20 and 3D structured light scanning 8. However, the designs only help to guide the complex geometries of the scaffolds.

A peripheral nerve consists of motor and sensory axons bundled together by support tissue into an anatomically defined trunk. The endoneurium surrounds individual axons and their Schwann cell sheaths. Next, the perineurium surrounds groups of axons to form fascicles. Finally, the epineurium binds individual nerve fascicles into a nerve trunk. Connective tissue is located between fascicles and includes a vascularized system for providing nutrition. In the 3D structure, the internal structures of nerve fascicles are complicated, and the relative position and number of nerve fascicles can change within a very short distance 25. Therefore, long nerve defects are always found to be mismatched and the degree of function recovery is low. Clinical evidence suggests that nanotopography and microtopography incorporated into scaffolds does not merely improve peripheral nerve regeneration, but is in fact a prerequisite for meaningful restoration of nerve function. Thus, we designed this study to verify the role of the precise matching of fascicles in effective recovery of nerve function. In addition, we provide a theoretical basis for future TENG designs on whether fascicles pathways need to be included to repair long nerve defects.

In our study, rat sciatic nerve defects measuring 15 mm in length were selected for reconstruction with various end-to-end neurorrhaphy formations. The effectiveness of regeneration for the 100%FM, 50%FM and 0%FM groups was evaluated at 6 weeks after surgery. The recovery of motor function in the injured hindlimb showed that the repair in the 100%FM group was better than that in the 50%FM and 0%FM groups, which was indexed by SFI values. The 100%FM group was confirmed to provide better support for nerve regeneration through histological evaluation. More axons successfully crossed the repair site from the proximal to the distal segment in the 100%FM group. The 100%FM group showed better reconstruction of the regenerated nerve and the gastrocnemius target muscle than the other groups according to both qualitative and quantitative assessments. These data indicate that the precise matching of peripheral nerve fascicles is useful in promoting effective nerve function recovery.

To our knowledge, this is the first study to report that precisely matching fascicles can enhance the repair capacity of PNIs in all autologous nerve grafts. The 100%FM group showed promising results for stimulating myelinated axon regeneration, rescuing muscle atrophy and quickly restoring nerve function among the three groups. Nanotopography and microtopography, such as the endoneurium, the perineurium and the epineurium, are very important physical guide elements incorporated into scaffold design that may play a significant role in long nerve regeneration. FM autologous nerve grafts as tissue-engineered scaffolds represent a promising treatment for the repair of large peripheral nerve defects and the promotion of nerve regeneration, providing a theoretical basis for future TENG designs.
